# Recreational drugs repurposed for medicinal use—cannabis

**DOI:** 10.1017/S109285292500032X

**Published:** 2025-04-25

**Authors:** Mike Armour, Justin Sinclair, Hannah Adler

**Affiliations:** 1 NICM Health Research Institute, Western Sydney University, Sydney, Australia; 2 Centre for Social and Cultural Research, Griffith University, Queensland, Australia

**Keywords:** Cannabis, pain, inflammation, cannabinoid, endocannabinoid, cannabidiol, tetrahydrocannabinol, marijuana

## Abstract

Cannabis has a long history as a medicine and was a part of medical practice until the late 19th century. The discovery of cannabidiol (CBD) and ∆9-tetrahydrocannabinol (THC) in the mid-20th century, and then the various components of the endocannabinoid system (ECS) over the following decades has again brought cannabis back into the public eye as a potential therapeutic agent. At present, cannabis is being used in the community across the world for both recreational and medical purposes. In the case of medical usage, it may be prescribed by a medical doctor or purchased either legally or illicitly for medical purposes such as symptom relief. Evidence for cannabis as a medicine is still an emerging field, and while potential mechanisms of action for a variety of conditions have been elucidated, including cancer, epilepsy, and chronic pain, high-quality randomized controlled trials in humans are still lacking. Despite popular beliefs, cannabis, like all other medicines, has potential benefits and harms, and long-term consumption of cannabis, even for medical reasons, may not be risk-free. In addition, consumption via modes of administration such as smoking or using a bong may increase the risk of negative health outcomes.

## Introduction

### A brief overview of the botany of cannabis

The Cannabaceae family (Order Rosales) is a small family of flowering plants currently encompassing 10 genera and 170 different species.^1^ Of these, the *Cannabis* genus has been of significant socio-cultural, entheogenic, and medicinal importance since antiquity, with additional utilization as a food stuff, textile, and cordage.^2^ Cannabis is associated with three species of flowering plants: Sativa, Indica, and Ruderalis.^3^ While historically contentious, the categorization of cannabis, aside from the formal botanical nomenclature classification,^4^ faces ongoing challenges of overcoming the inconsistent application of “folk-taxonomy,” observed with the overuse of the terms “Sativa” or “Indica”.^1^ While these terms are ubiquitously applied across the medicinal, legal adult-use, and illicit spheres, such terminology is pointless given the amount of cannabis hybridization and interbreeding that has taken place,^1^ rendering the terms as having little or no practical relevance. For the purposes of this chapter, cannabis is perhaps best characterized predominantly based on its phytochemistry, and the cultonomic categorisation laid down by the International Code of Nomenclature for Cultivated Plants (ICNCP), which recognizes cannabis cultivars by their economically important characteristics.^5^

Botanically, cannabis is characteristically dicotyledonous (possesses a tap root), annual, dioecious (male and female reproductive parts are on separate plants) and herbaceous,^5^ with the primary product being the dried female inflorescence (cluster of flowers).^6^ The unfertilized female plant produces the highest amounts of cannabinoids and terpenes, as secondary metabolite production is deprioritised should fertilization occur. On these flowers, specifically the calyces and bracts, and to a lesser extent other structures such as flower leaves (that is sugar leaves) and stems, are the main morphological structures of pharmacological interest—the trichomes. Trichomes (From Greek *trikho* meaning ‘hair’) are small, unicellular or multicellular filamentous appendages that grow outward from the epidermis and serve a number of functions, including protecting the plant from ultraviolet irradiation, pathogens, pest deterrence, excessive transpiration, and ruminant herbivores.^6^^,^^7^ Historically, these trichomes have been harvested, most commonly using fine mesh sieves, and compressed into a resinous material popular in illicit trade known as hashish (aka hash), representing a more potent format for consumption than dried flower alone. Cannabis has two predominant trichome types: glandular, cannabinoid-producing-trichomes (that is capitate-stalked glandular trichomes) and non-glandular, non-cannabinoid producing trichomes.^8^ The capitate glandular trichomes of cannabis are the main site for cannabinoid and terpene/terpenoid production and storage.^9^^,^^10^

### The history of cannabis as a medicine from pre-history to present day

While in the twenty-first century the use of cannabis for medicinal purposes is seeing a resurgence worldwide, cannabis has a long and rich history.^11^ It is “*certainly among the most ancient plants that have been grown and exploited by humankind for its countless properties and uses as a fiber, food, and drug plant”.* ^12^ The use of cannabis is suggested to predate human evolution,^13^ and paleobotanical studies argue that it was present during the Holocene epoch roughly 11,700 years ago.^12^ Central Asia has been suggested as the place in which cannabis is indigenous,^14^ with archaeological evidence placing the plant in China 6,000 years ago during the Neolithic period.^15^ While the first documented use of cannabis as a medicine remains contested, some suggest it dates back to 4000 BC^12^ where it was utilized as an anesthetic during surgery, and elixirs were incorporated into certain Doaist religious ceremonies.^16^ Others have argued that the earliest records of medicinal cannabis date back to 2800 BC, where it was listed by Chinese Emperor Shén Nóng in his list of therapeutic indications.^17^ It has also been suggested by Li^15^ that the first documented medicinal use of cannabis can be found in an herbal text of the 2nd century AD (Book of Odes), which is filled with oral traditions which were passed down from prehistoric times. However, cannabis is often known for its place in traditional Indian medicine, as India developed a long and continuing tradition of cannabis cultivation for medicinal and religious use.^12^^,^^16^ While cannabis also has a long and rich history globally,^18^ it is this use in Indian medicine that saw it be introduced to Western pharmacopeia’s in the nineteenth century.

Dr William Brooke O’Shaughnessy, a physician and professor of chemistry and *Materia Medica*, is largely credited as the first to apply a Western experimental method in studying cannabis during his time in India in the 1830s.^11^^,^^19^ He noted that this cannabis, which was described as Indian cannabis (*Cannabis indica*), was a different variety from the cannabis being used in Europe for the process of fiber manufacturing, which was *Cannabis sativa.* ^12^ As explained by Kalant (2001), he observed the use of cannabis *“for the treatment of spastic and convulsive disorders such as ëhydrophobiaí (rabies), tetanus, cholera, and delirium tremens.”* He sent supplies of the plant to London for analysis and clinical study,^11^ and when returning to England in 1841, he brought seeds of *C. indica* with him for investigation by the Pharmaceutical Society.^20^ By the end of the nineteenth century, cannabis had been adopted into British (and subsequently, Australian), and American pharmacopoeias and was identified in the Lancet medical journal by the physician of Queen Victoria, Sir J. Russel Reynolds, as a useful analgesic. During this time, cannabis was used throughout Europe and English-speaking countries for many different treatments and remedies.^12^^,^^20^ This was due to the efforts of O’Shaughnessy and others, such as French psychiatrist Jacques-Joseph Moreau and Baron Antoine de Sacy, who were prominent figures in the study of “hashish”.^20^ However, the approach to drugs as being a personal choice outside of the scope of government intervention,^21^ began to shift toward the end of the nineteenth century due to temperance movements. These movements not only lobbied effectively for increased controls regarding drugs but also framed them as problematic and requiring regulation,^22^ inevitably affecting the legitimacy of cannabis as a medicine.

This delegitimization was coupled with the rise of orthodox drugs, as more standardized, synthetic drugs such as opioids became the focus of biomedicine,^11^ while cannabis became associated with “marijuana” through the political campaign Reefer Madness. Through cinema and newspaper reports, this campaign framed cannabis (‘marijuana’) as a dangerous drug used by minorities rather than a medicine with a rich cultural history—demonising both cannabis and those who used it.^19^^,^^23^ The Commissioner of the Federal Bureau of Narcotics at the time, Harry J. Anslinger, attempted to associate cannabis with psychosis, mental deterioration, addiction, and violent crimes.^19^ This era of prohibition led to cannabis being removed from the British Pharmacopeia in 1932 and to the introduction of the United States Marijuana Tax Act of 1937. This latter act was opposed by the American Medical Association at the time, who stated “*that legislation should not prohibit medicinal use and scientific investigation”.* ^19^ Despite these efforts, it was removed from the American Pharmacopeia in 1942, and penalties for the possession of cannabis increased in 1951 and 1956.^24^ By the 1970s, and largely due to the rewriting of federal drug laws by President Richard Nixon, cannabis was placed as a Schedule 1 substance under the Controlled Substance Act of 1970. This meant cannabis was considered of high abuse potential with no medicinal value,^25^and was in the same schedule as heroin and lysergic acid diethylamide (LSD).^19^

However, due to a rise in scientific interest, the twentieth century saw cannabis be once again considered a medicine. It is suggested that this interest in medicinal cannabis was a collateral effect of the opioid abuse epidemic and increased research from Israel.^24^ In 1964, the chemical structure responsible for the intoxicating effects of cannabis was reported by two Israeli researchers, Mechoulam and Gaoni, with this discovery being the gateway for their research into the endocannabinoid system.^17^ Despite the prohibitive scheduling of cannabis in America, this research sparked a conversation about the medicinal use of cannabis around the globe. Thus, in 1996, the 1996 Compassionate Use Act was passed in California, and it became the first state in America to allow for the use of medicinal cannabis.^19^ Since this time, both medicinal and recreational cannabis have been made available in a variety of states in America and the District of Columbia, yet it remains prohibited federally. Outside of America, Uruguay became the first country in the world to legalize recreational cannabis in 2013, and other countries such as the Netherlands and Canada allow for both medicinal and recreational use, whereas others such as Australia allow just medicinal use, facilitating a slow return to the acknowledgment of medicinal cannabis and its rich history.

### The endocannabinoid system & impact of cannabis research on science

Cannabidiol (CBD) was first discovered in 1940 by Adams and colleagues^26^ but was not fully elucidated until 1963 by Mechoulam and Shvo^27^ through advances in separation chemistry. A year later, ∆^9^-tetrahydrocannabinol (THC), the primary cannabinoid responsible for the intoxicating effects of cannabis, was also discovered.^28^ With these discoveries commenced a renewed scientific interest in cannabis research, which over 20 years later would discover specific cannabinoid receptors; the cannabinoid 1 receptor (CB1) being discovered in 1988,^29^ and the CB2 receptor being identified in 1993,^30^ both belonging to the family of 7-transmembrane G _i/o_ protein-coupled receptors (GPCR).^31^ CB1 receptors, encoded by the CNR1 gene, are ubiquitously distributed throughout the central nervous system (CNS), where they are the most abundant GPCR, far exceeding those for the neurotransmitters (NTs) they modulates,^32^ being highly expressed in the hippocampus, basal ganglia, and cerebellum; moderately expressed in the cerebral cortex, amygdala, hypothalamus, and dorsal horn of the spinal cord; and minimally expressed in the thalamus.^33^^–^^35^ CB1 receptors are highly expressed on presynaptic terminals, whereby they mediate retrograde signaling of endocannabinoids and their subsequent ability to inhibit synaptic transmission (suppressing the release of a range of NTs), but are also expressed to a lesser extent in astrocytes, microglia, and oligodendrocytes.^34^ Aside from CNS distribution, the CB1 receptor is also abundant across the peripheral nervous system (PNS) and is found in the gastrointestinal tract, liver, skeletal muscles, pancreas, lungs, bladder, adrenal glands, and cardiovascular and reproductive systems.^34^^,^^36^^,^^37^ In contrast, the CB2 receptor is expressed at much lower levels in the CNS compared to CB1,^38^ but plays a crucial role in CNS immune response by regulating microglial activities,^39^ and being highly inducible (up to 100 fold expression) following inflammation or tissue injury.^40^^,^^41^ CB2 receptor presence has been noted in the tonsils, bone marrow, pancreas, spleen, mast cells, and peripheral blood leukocytes,^42^ and is primarily expressed when and where there is active inflammation. Unlike CB1, the CB2 receptor appears to be devoid of addiction liability or psychotropic effects and is a promising therapeutic target in neuropathic pain and neuroinflammatory conditions.^40^ Aside from the roles of CB1 and CB2, numerous other receptors have been implicated as putative endocannabinoid receptors, such as G-Protein Receptor (GPR) 55,^43^ GPR119^44^ and GPR18,^45^ further demonstrating the complexity of the endocannabinoid system and the importance of continuing research to fully elucidate its wide-ranging spectrum of biological activities.

Concurrent research then solved the next piece of the physiological puzzle—identifying the endogenous ligands that bound to these cannabinoid receptors, with anandamide (N-arachidonoylethanolamide) being discovered in 1992^46^ and 2-AG (AEA; 2-arachidonoylglycerol) in 1995.^47^^,^^48^ Both AEA and 2-AG are categorized as bioactive lipids (arachidonic acid derivatives), belonging to the subclasses of N-acylethanolamines and monoacylglercerols, respectively,^49^ and are synthesized on demand from cell membrane phospholipids, a stark difference from classical NTs and neuropeptides, which are stored in intracellular vesicles. Post-production, these endocannabinoids are subsequently released into the synaptic cleft from the postsynaptic terminal, where they bind to cannabinoid receptors on the presynaptic membrane^49^; This activity regulates synaptic neurotransmission in a retrograde fashion, controlling both inhibitory and excitatory inputs *via* inhibiting N- and P/Q-type Ca2+ channels and activating K+ channels.^49^^,^^50^ AEA exerts partial agonism (akin to THC) at cannabinoid receptors, activates transient receptor potential vanilloid 1 receptors (TRPV1),^51^ and was named anandamide from the Sanskrit word “*Ananda”* meaning bliss—a reference to its ability to mimic the psychotropic effects of THC.^52^ In contrast, 2-AG exerts full agonism at both cannabinoid receptors and is considered a fast retrograde synaptic messenger. Aside from these two primary endocannabinoids, other lipids have been identified with “endocannabinoid-like” activity, such as 2-arachidonylglyceryl ether (2-AGE, noladin), O-arachidonylethanolamine (virodhamine), N-palmitoylethanolamide (PEA), *N*-oleoylethanolamine (OEA), *N*-stearoylethanolamine (SEA), and N-arachidonyldopamine (NADA)^49^^,^^52^; however. their function(s) are currently unclear.

Finally, the enzymes involved in the synthesis and catabolism of the endocannabinoids were the last piece to fall into place, such as fatty acid amide hydrolase (FAAH), which is responsible for anandamide degradation, and monoacylglycerol lipase (MAGL), which degrades 2-AG.^31^ Numerous other enzymes have since been discovered that play an integral role in endocannabinoid biosynthesis and degradation, such as the α/β-hydrolase domain (ABHD) enzymes, such as ABHD6 and ABHD12, which collectively contribute up to 15% of 2-AG hydrolysis^53^^,^^54^ Interested readers will find a comprehensive understanding of cannabinoid receptors, their ligands, and associated enzymatic synthesis and degradation pathways in the following articles.^49^^,^^55^^–^^57^

The discovery of the cannabinoids within cannabis led to the systematic unearthing of previously unknown cannabinoid receptors, endogenous ligands, and the enzymes involved in ligand synthesis and catabolism, resulting in what is now known as the Endocannabinoid System (ECS). The ECS plays an important role in regulating a broad list of physiological homeostatic processes such as digestion, immune function, nociception (that is pain), neural development, learning, memory, metabolism, inflammation, appetite regulation, cardiovascular and respiratory function, and sleep-wake cycles,^31^^,^^58^ representing an entire neuromodulatory system previously unknown to humanity and which is likely one of the most significant medical discoveries of the last 60 years, providing a new understanding of previously unknown dysfunctions in various diseases such as endometriosis, as well as potential therapeutic targets to treat a wide range of conditions.

### Phytochemistry and pharmacology

Currently, there are believed to be over 750 different secondary metabolites^5^ identified across the different *Cannabis* varieties, including the cannabinoids and terpenes/terpenoids, as well as simple phenolic glycosides, flavonoids, aldehydes, ketones, esters, phytosterols, coumarins, simple phenols, alkaloids, and fatty acids.^5^^,^^59^ Many of these compounds have not been investigated for pharmacological activity. This complex matrix of phytochemical constituents makes it challenging for researchers to understand the complete range of pharmacological activity associated with many plant medicines but is also possibly why cannabis is being utilized across a wide range of symptoms and clinical indications due to its extensive multi-target activity.

#### Cannabinoids

The term cannabinoid is wide-ranging and is used to describe synthetic cannabinoids, endocannabinoids (e.g. N-arachidonoylethanolamine and 2-Arachidonoylglycerol) and phytocannabinoids (naturally occurring cannabinoids in plants);^60^^,^^61^ all of which interact with cannabinoid (that is CB1, CB2) or other receptor types. Generally, cannabinoids are highly lipophilic, able to permeate cell membranes and cross the blood–brain barrier (whether *via* ingestion or inhalation),^5^ which offers both positive and negative attributes when viewed as a medicinal agent.

The phytocannabinoids are a unique class of terpeno-phenolic compounds, and to date, over 144 different cannabinoids have been identified using high-performance liquid chromatography (HPLC), mass spectrometry (MS) and other analytical methods,^62^ with some being artifacts of analysis. The terpeno-phenolic cannabinoids are derived from the enzymatic condensation of both a terpene moiety (e.g. geranyl pyrophosphate) and a phenolic moiety (typically olivetolic acid or diverinic acid),^63^ which produces the progenitor compound cannabigerolic acid (CBGA), the compound from which all other cannabinoid acids are derived.

In the living plant, phytocannabinoids exist in acidic form, with a carboxylic acid (COOH) group attached to the phenolic ring.^63^ Removal of the carboxylic acid (that is decarboxylation) is required to transform the acidic form into the neutral analog, usually through exposure to heat or drying, or to a lesser extent, light. Examples of these phytocannabinoid acids include cannabidiolic acid (CBDA), ∆^9^-tetrahydrocannabinolic acid (THCA), and cannabigerolic acid (CBGA), all of which transform through the process of decarboxylation to the neutral analogs cannabidiol (CBD), ∆^9^-tetrahydrocannabinol (THC) and cannabigerol (CBG), respectively. Aside from the presence of the carboxylic acid group, another unique aspect of the cannabinoid molecule is the polyketide chain in the *meta* position, which is typically pentyl (5-carbons), but can also exhibit propyl (3-carbons) or methyl (CH_3_), side chains.^63^ For a comprehensive analysis of phytocannabinoid chemistry and biogenesis, the reader is directed to the works of Hanus and colleagues.^64^

The phytocannabinoids are typically divided into 11 subclasses based on their chemical structure, which comprises precursors, byproducts, and degradation products, and includes ∆^9^-THC, ∆^8^^-^THC, CBG, CBD, cannabinol (CBN), cannabichromene (CBC), cannabicyclol (CBL), cannabielsoin (CBE), cannabinodiol (CBND), cannabitriol (CBT), and miscellaneous types.^62^ Of these, CBD and THC have received the vast majority of research focus, and due to this, they form the basis for formulation standardization for the majority of medicinal cannabis products currently utilized for patient care and symptom management worldwide.

#### *∆* ^9^ *-Tetrahydrocannabinol (THC)*

Cannabis is the most cultivated, trafficked, and consumed illicit drug worldwide, and accounts for half of all drug seizures internationally.^65^ This is due to the content of THC, the main intoxicating/psychoactive phytocannabinoid, which, through selective breeding programs, is the most abundant cannabinoid found across the hundreds of different cannabis cultivars (sometimes incorrectly referred to as strains) observed across illicit, legal adult-use, and medical domains.

THC exhibits high lipid solubility and is a partial agonist at both the CB1 (K_i_ = 10 nM) and CB2 (K_i_ = 24 nM) receptors,^33^ binding with relatively high affinity and expressing similarity to the endogenous cannabinoid anandamide.^66^^,^^67^ The interaction between THC and CB1 receptors results in a downregulation of the secondary messenger cAMP by inhibition of adenylate cyclase, resulting in the intoxicating effects (euphoria, relaxation, analgesia) associated with THC.^33^ Aside from cannabinoid receptor interaction, other receptor-mediated modulation includes positive allosteric modulation of glycine receptors, antagonism of the TRPM8 ion channel, agonism at the PPAR-γ nuclear receptor, agonism of TRPV2, TRPV3, TRPV4, and TRPA1 ion channels, and negative allosteric modulation of serotonergic (5HT3) receptors as well as *μ* and *δ* -opioid receptors.^68^^,^^69^ THC also exhibits partial agonistic activity at the orphan GPR18 and GPR55 receptors,^69^ which have been proposed as putative cannabinoid receptors.^70^

THC has a wide range of pharmacological activity described in the literature, including analgesic,^71^^,^^72^ anti-inflammatory, antioxidant,^73^ hypnotic,^74^ neuroprotective,^75^ bronchodilatory,^76^ anticancer^77^^–^^83^ appetite stimulant, and antiemetic actions.^9^^,^^84^ Such pharmacological activity makes it clinically useful for many different indications, including neuropathic pain,^85^^,^^86^ migraine,^87^ cancer pain,^88^ chemotherapy-induced nausea and vomiting,^89^ and chronic pain.^90^^,^^91^ Additionally, THC has potential in the symptomatic management of various neurological disorders such as multiple sclerosis (that is muscle spasticity)^92^ and Alzheimer’s disease,^93^ and can lower intraocular pressure in glaucoma.^94^^,^^95^

THC bioavailability and pharmacokinetics, like all cannabinoids, are primarily dependent on the route of administration (i.e., dosage format) and formulation used.^96^ When inhaled, the bioavailability of THC has been reported at 10–35%,^97^^,^^98^ with such variability being in part due to intra- and inter-subject variability across factors such as spacing of inhalations, hold time, the number and duration of inhalations, and inhalation volume.^99^ Cannabinoids administered *via* inhalation display comparable pharmacokinetics to intravenous administration,^96^ with peak plasma concentration attained within 3–10 minutes,^97^ and greater concentrations achieved relative to oral ingestion, due largely to inhalation avoiding substantive first-pass metabolism.^96^ Along with a fast onset of action, the duration of effects of inhaled consumption typically ranges between 2 and 4 hours.^100^ Additionally, a third to half of cannabinoids present in cannabis material are pyrolyzed during the combustive process of smoking,^99^ albeit this is not a concern for inhalation *via* vaporization due to lower temperature utilisation. Furthermore, it is posited that vaporisation reduces risks associated with combusted inhalation due to the reduction in exposure to pyrolytic compounds,^101^ and is comparable in pharmacokinetics to smoked cannabis,^102^ so it may be a safer route of administration when fast onset of pharmacological activity is required.

THC oral absorption is poor, slow, and unpredictable, with oral bioavailability of THC food products (i.e., edibles) ranging between 6% ± 3%, and 10–20% in cannabis oral extracts.^103^ Due to extensive first-pass hepatic metabolism, delays in onset of pharmacological effects compared to inhaled formats are noted, with maximal plasma concentrations of THC usually occurring between 60 and 120 minutes,^96^^,^^97^ with some studies showing maximal plasma concentrations as late as 4–6 hours. Despite a slow onset of effect, oral dosage forms confer a longer duration of effect, ranging between 6 and 8 hours,^100^ so are useful when longer-lasting symptomatic relief may be required.

THC is rapidly distributed throughout well-vascularized tissues and organs, predominantly the lungs, heart, brain, and liver,^96^ but also the kidney, thyroid, and jejunum.^97^ Approximately 90% of THC in blood is distributed *via* plasma, with the remaining 10% to red blood cells, with 95–99% of plasma THC being bound to plasma proteins such as lipoproteins and, to a lesser extent, albumin.^97^ Similar to other cannabinoids, fat is also a site for THC accumulation, particularly with chronic administration. As such, THC can diffuse out of fat and into blood days to weeks after cessation of dosing, a cause for concern with relation to drug driving laws in some jurisdictions where THC detection *via* oral swab is an offense, even if cannabis is medically prescribed.^104^

THC metabolism is primarily hepatic, *via* the isoenzymes CYP2C9, CYP2C19, and CYP3A4.^96^ THC is predominantly metabolized to 11-hydroxy-THC (11-OH-THC), a psychoactive metabolite,^105^ and 11-carboxy-THC (11-COOH-THC), which after glucuronidation processes, are excreted in feces (65%) and urine (20%).^96^^,^^98^ Extra-hepatic tissues (i.e., that express CYP450 enzymes), such as the intestines and brain, can also take part in metabolism.^96^^,^^99^ Furthermore, as THC is lipophilic, it can cross the placenta and has been found in expressed breast milk,^96^ an important clinical consideration given the impact of THC on the developing infant is not clear.

The elimination of THC is difficult to calculate and can vary considerably amongst individuals, with the main reason being the slow rediffusion of THC from body fat and other tissues back into the circulatory system.^97^ Notwithstanding, THC plasma half-life ranges between 1–3 days in infrequent consumers to 5–13 days in chronic consumers.^98^

#### Cannabidiol (CBD)

CBD is a non-intoxicating phytocannabinoid with a well-established safety profile, exhibiting no risk indicative of addiction or dependence potential.^106^ Interestingly, CBD displays little affinity for the CB1 or CB2 receptor, with no direct interaction with the orthosteric binding site being evident,^107^ however, it has been proposed as a negative allosteric modulator of the CB1 receptor.^108^ Notwithstanding, CBD has had over 65 molecular targets identified,^107^ distinct from the ECS, and is a complex, multi-target molecule. CBD is an agonist for the serotonin (5HT_1A_)^109^ receptor, a partial agonist of 5HT_2A_ and non-competitive antagonist of 5HT_3A._^69^ Additionally, CBD is a full agonist at TRPV1^110^ and activates TRPV2, TRPV3, and TRPV4 ^107^, and has also been noted as enhancing the activity of α-1 and α-3 glycine receptors and PPAR-γ.^69^ CBD has also been found to be an antagonist of GPR55 and GPR18 and an agonist of TRPA1.^111^ Furthermore, CBD is also an allosteric modulator of *mu* and *delta*-opioid receptors,^112^ and can increase the levels of anandamide due to an inhibitory effect on FAAH.^69^ For a more detailed summary of the range of CBD targets, the reader is directed to the works of Mlost and colleagues.^113^

Much akin to THC, CBD is highly lipophilic and possesses poor bioavailability, with some studies suggesting this can be as low as 6%.^96^ Conversely, 4–5 fold increases in CBD absorption have been noted when ingested orally with a meal rich in fats.^114^ CBD exhibits >95% protein binding capability,^115^ which is an important clinical consideration in those impacted by low albumin levels or liver disease. When inhaled, CBD has an average systemic bioavailability of 31%^97^ and shares a similar concentration-time profile as THC.^96^ Upon oral ingestion, CBD is subject to first-pass hepatic metabolism, with a peak concentration generally being reached within 2–3 hours. The C_MAX_ and area under the curve (AUC) after oral ingestion are dose dependent, with a dose of 10 mg of CBD exhibiting a mean C_MAX_ of 2.47 ng.mL at 1.27 hours, compared to a dosage of 800 mg of CBD, which exhibited a C_MAX_ of 77.9 ng.mL, with a mean T_MAX_ of 3 hours.^116^ The mean half-life (t-_1/2_) of 10 mg and 20 mg doses (administered orally) of CBD has been reported at 1.09 and 1.97 hours, respectively, and 3 hours post-smoking.^116^

Similar to THC, CBD distribution is noted to rapidly distribute through most tissues, particularly those that are well vascularized such as the lungs, heart, brain, and liver, and due to its lipophilic nature it has also been noted to accumulate in adipose tissue, particularly after long-term use.^96^ The metabolism of oral CBD involves extensive hepatic involvement, mainly through the cytochrome P450 system, but can also impact drug excretion through the p-glycoprotein drug transporter.^99^^,^^117^ Specific to the former system of metabolism, specific isoenzymes involved in CBD metabolism include CYP2C19, CYP3A4, CYP1A1, CYP1A2, CYP2C9, and CYP2D6.^96^^,^^118^ First-pass hepatic metabolism causes the formation of numerous metabolites, most notably 7-hydroxy-cannabidiol (7-OH-CBD) which occurs *via* hydroxylation reaction. Due to the involvement of numerous isoenzymes, CBD has the potential to potentially impact the way certain pharmaceutical medications are metabolized and therefore impact their serum levels and subsequent therapeutic efficacy.

With a broad array of interactivity at numerous receptors, CBD has a wide biochemical scope, with a therapeutic potential equal or greater to that of THC. CBD has a well-researched anti-inflammatory activity, it being suggested to enhance adenosine signalling by inhibiting adenosine inactivation.^119^ CBD also exhibits significant neuroprotective,^120^ antioxidant^121^ immunomodulatory,^120^ antipsychotic,^5^ anxiolytic,^122^ antidepressant,^123^ anti-angiogenic,^124^ hypnotic, sedative, analgesic, and antiemetic activity,^5^ all of which are of potential benefit to multiple chronic diseases.

Common side effects that have been recorded in the literature specific to CBD use in the clinical setting are changes in appetite, diarrhea, sedation, tiredness, sleep disturbance, anemia, changes in transaminase levels (elevation) or infection.^117^^,^^125^ Dose appears to play an important role in both drug interactions and side effects/adverse events associated with cannabidiol.

### Minor cannabinoids

Aside from THC and CBD, numerous minor cannabinoids are starting to garner research interest and are divided into neutral, acidic, and varinic phytocannabinoids.^126^ These include CBG, CBN, CBC, THCA, CBGA, tetrahydrocannabivarin (THCV), and cannabidivarin (CBDV),^126^ albeit this list is not exhaustive.

#### Cannabigerol (CBG)

Like CBD, CBG is a non-intoxicating cannabinoid which was first isolated in 1964 and is found more prevalently in commercial hemp varieties.^61^ The acidic form of CBG, CBGA, is the major precursor compound for other cannabinoids, including CBD, CBC, and THC.^127^ While there is conflicting data, the best evidence suggests that CBG exhibits weak partial agonist activity at the CB1 and CB2 receptors, is a GABA uptake inhibitor, a potent TRPM8 antagonist, an agonist of α2-adrenergic receptors, and works as a 5HT_1A_ antagonist.^5^^,^^61^^,^^126^ Additionally, CBG activates TRPV1, TRPV2, TRPV3, TRPV4, and TRPA1 channels; binds to and activates PPARγ; and is a potent competitive inhibitor of anandamide.^126^^,^^128^ While the research on CBG is in its relative infancy compared to THC, there is some data on the pharmacokinetics of CBG. CBG has a half-life of 2–6 hours after oral administration, and post-inhalation is present in plasma within minutes and reaches T_max_ in 0.17 hours, followed by a rapid decrease in concentration (similar to THC and CBD).^128^ CBG is primarily metabolized by the CYP2J2, producing monohydroxy compounds, and is excreted in conjugated form through urine.^128^ As another multi-target cannabinoid, CBG has demonstrated numerous pharmacological effects, including antioxidant, anti-inflammatory, neuroprotective, antitumor, appetite-stimulating, and antimicrobial activities.^61^^,^^128^^,^^129^

#### Cannabinol (CBN)

The non-intoxicating cannabinoid CBN was the first cannabinoid isolated from cannabis in 1896,^64^ and its structure was reported in 1940.^130^ Unlike other cannabinoids, which have been identified in other plants and fungi, CBN has as yet only been found in cannabis.^131^ In contrast to the other cannabinoid acids and their derivation from CBGA, a biosynthetic pathway for cannabinolic acid has not yet been identified.^126^^,^^132^ As such, CBN is seen as an artifact of degradation from THC (*via* aromatisation) generally mediated by heat, light, and oxygen,^132^^,^^133^ and may be found in higher concentrations in aged cannabis products as levels of THC decrease. CBN exhibits low binding affinities for the CB1 and CB2 receptors comparative to THC,^126^ and is an agonist at TRPV1-TRPV4 channels, a potent agonist of TRPA1, and inhibits activation of TRPM8 as a potent antagonist.^126^^,^^132^ While not investigated extensively pre-clinically or clinically, evidence suggests that CBN exhibits analgesic, anti-inflammatory, antibacterial, orexigenic, hypnotic, anticancer, and potential neuroprotective properties.^126^^,^^131^^,^^132^

#### Cannabichromene (CBC)

Along with THC, CBD, and CBN, CBC is another phytocannabinoid prevalent in various cannabis varieties.^134^ Like CBD and THC, CBC is synthesized from CBGA and all share a common 3-pentylphenol ring.^135^ The structure of CBC was not determined until 1966,^136^ and its concentration in the plant is generally low (0.2–0.3% dry weight),^61^ albeit this is dependent on chemotype. A non-intoxicating cannabinoid, CBC is a potent activator of TRPA1 channels, a weak inhibitor of monoacylglycerol lipase (MAGL), activates TRPV3 and TRPV4, and displays similar affinities for the CB1 and CB2 receptors, causing receptor-mediated decreases in cellular cAMP levels.^126^^,^^134^^,^^137^ Pharmacological activity ascribed to CBC includes antimicrobial, analgesic, antiproliferative, potential neuroprotective, and anti-inflammatory effects.^5^^,^^61^^,^^126^

#### ∆^8^-Tetrahydrocannabinol (∆^8^-THC)

Unlike many of the other phytocannabinoids, ∆^8^-THC is an intoxicating cannabinoid present in much smaller concentrations in the cannabis plant than ∆^9^-THC.^138^ Due to this, many ∆^8^-THC products being used by consumers, particularly in North America, are obtained *via* the cyclization (acid-catalysed conversion) of CBD.^139^ ∆^8^-THC is a double bond isomer of ∆^9^-THC, differing in molecular structure from ∆^9^-THC with the position of the double bond being between carbon atoms 8 and 9, whereas ∆^9^-THC is between 9 and 10.^140^ ∆^8^-THC was first derived from the cyclization of CBD and found to be psychoactive,^141^ but due to its differing structure, is not as potent as ∆^9^-THC as it has lower affinity for CB1 receptors.^140^^,^^142^ Similar to ∆^9^-THC, ∆^8^-THC is a partial agonist of CB1 and CB2 receptors, but unlike ∆^9^-THC, it is far more chemically stable, which, coupled with a lower intoxication profile, makes it an attractive compound for further research.^138^ However, 104 reports of adverse events related to ∆^8^-THC have been reported to the Food and Drug Administration (FDA) between 2020 and 2022,^143^ and are similar to acute cannabis intoxication seen in ∆^9^-THC, which is important for clinician awareness, particularly given that a lack of regulation of ∆^8^-THC products across the USA makes this a more challenging issue.^139^ Pharmacological activities associated with ∆8-THC include analgesia, antidepressant, lowering intraocular pressure, anticancer and decreased seizure activity.^144^^–^^146^

### Terpenes and terpenoids

Much akin to the terpeno-phenolic cannabinoids, terpenes and terpenoids are another phytochemical class manufactured within the glandular trichomes of cannabis and form one of the largest groups of plant chemicals, with between 15,000 and 20,000 being fully characterized, and over 200 being reported across cannabis varieties.^9^^,^^147^ Terpenes and terpenoids are essential oil components that are volatile organic compounds commonly associated with the different smells associated with plants,^148^ and serve an important protective role as secondary plant metabolites that can exhibit antimicrobial and antifeedant properties. Specific to cannabis, the glandular trichomes, which house these volatile compounds, are believed to be a plant defense mechanism, particularly against light stress,^149^ but also have antifeedant, antimicrobial, and insect-repellent activity.^9^

#### Terpenes

Terpenes, often also referred to as isoprenoids, are characterized as simple hydrocarbon compounds based on 5-carbon (C5) isoprene units, with monoterpenes (C10) and sesquiterpenes (C15) being the predominant components of essential oils,^150^ and the main components with noted pharmacological activity across cannabis varieties. Monoterpenes are the most prevalent component in essential oils, followed by sesquiterpenes, the former succumbing to higher loss with drying, heat, and storage than the latter.^150^ Acyclic monoterpenes such as β-myrcene, bicyclic monoterpenes such as *a*-pinene, and monocyclic monoterpenes such as limonene have a broad range of pharmacological activities.^150^ β-myrcene is an agonist at *a*2-adrenergic receptors and TRPV1,^151^ and has reported analgesic, anti-inflammatory, antibacterial, and sedative pharmacological effects, the latter being described as a “couch-lock” effect when in concentrations over 0.5% in combination with THC.^9^^,^^152^^–^^154^ Common in conifers, *a*-pinene is one of the most common terpenes in nature and has noted anti-inflammatory, bronchodilatory properties and inhibits the activity of acetylcholinesterase in the brain, potentially aiding in memory and minimizing cognitive dysfunction observed with THC intoxication.^9^^,^^155^^,^^156^ Further research posits *a*-pinene possesses antimicrobial, antioxidant, and anti-allergic activity.^157^ Common to lemon and other citrus varieties, d-limonene has reported antibacterial, antifungal, insecticidal, anthelmintic, antioxidant, anti-inflammatory, neuroprotective, antiviral, and anxiolytic activities.^9^^,^^158^^,^^159^

β-caryophyllene (BCP) is one of the most commonly occurring sesquiterpenes found in cannabis, particularly post-decarboxylation, and exhibits a spicy, peppery aroma.^156^ BCP is a selective full agonist at the CB2 receptor, with some proposing BCP as a dietary phytocannabinoid.^9^^,^^160^ Additionally, BCP is an agonist at PPAR-γ and the toll-like receptor 4 (TLR4)/CD14/MD2 complex.^151^ BCP exhibits anti-inflammatory, gastroprotective, analgesic, anxiolytic, antibacterial, and antidepressant effects.^156^^,^^161^ Structurally similar to BCP, *a-*humulene (AKA *a*-caryophyllene) exhibits antibacterial, antifungal, antiparasitic, and anti-inflammatory activity.^162^

#### Terpenoids

Terpenoids are modified oxygen-containing terpenes with different functional groups,^150^^,^^161^ with at least 80 000 different compounds characterized.^163^ These terpenoids can be further divided into ketones, ethers, esters, aldehydes, alcohols, and phenols.^150^ Notable examples of monoterpene terpenoids include the acyclic linalool and geraniol, monocyclic monoterpenoids such as thymol, and bicyclic monoterpenoids thujone and cineole.^150^ Linalool, found in *Lavandula* (Lavender) species and certain cannabis varieties, has reported antidepressant activity *via* inhibition of serotonin reuptake,^164^^,^^165^ and also possesses antioxidant, anti-inflammatory, antimicrobial, and anxiolytic activities.^166^ Similar to linalool, thymol also possesses anti-inflammatory, antioxidant, and antimicrobial activity, as well as anticonvulsant, wound-healing and radioprotective actions.^167^

### Entourage effects

The concept of phytochemical synergy, whereby multiple phytochemicals, or herbal medicines, interact in dynamic and meaningful ways to augment or support absorption, reduce side effects, or increase therapeutic potency, is not a new concept to herbalists, having been discussed in formularies and pharmacopoeias since ancient times.^2^^,^^168^ Specific to cannabis, Ben-Shabat and colleagues coined the term “*entourage effect”* to describe the synergy/interactivity of endogenous fatty acid glycerol esters (which are pharmacologically inactive) enhancing 2-AG activity,^2^^,^^169^ and later, the possible synergistic or entourage-like activity between cannabinoids and terpenes was first posited by Russo.^9^ While research is ongoing into the possible synergistic relationships between various classes of compounds in cannabis, some authors have speculated whether the use of the term “*entourage effect”* is scientifically valid, as other natural plant-based products that are also composed of a broad spectrum of phytochemical compounds do not use such terms but rather traditional pharmacological terms such as synergistic, antagonistic, or additive effects.^170^

## Current evidence for medical benefit

While cannabis is being consumed by those in the community for a variety of medical conditions and has a long, traditional, and indigenous history as a medicine, there is currently a paucity of animal and human studies in most conditions. People with chronic conditions, or conditions where they do not feel that their current therapies are effective, often self-medicate with cannabis.^171^ Our focus in this article will cover several areas that have the most robust evidence, either positive or negative.

### Cancer

Cancer appears to demonstrate an upregulation of both CB receptors and endocannabinoids in tumors,^172^ suggesting a dysregulation of the ECS may be involved in cancer pathogenesis and progression, with different signaling pathways activated between healthy and malignant cells.^173^ There is a strong correlation between expression of CB receptors and increased malignancy/poorer prognosis in various types of cancers. Increased CB1 receptor expression has demonstrated worse prognosis across ovarian,^174^ pancreatic,^175^ prostate,^176^ and colorectal cancers,^177^ while increased CB2 receptor expression indicated a worse prognosis in breast cancer^178^ and squamous cell carcinoma.^179^ There are some exceptions to this; for example, non-small-cell lung cancer increased expression of CB1 and CB2 improved survival.^180^ In a similar fashion, there are often increased concentrations of endocannabinoids such as AEA and 2-AG in tumors when compared to surrounding healthy tissue.^181^ Therefore, it’s reasonable to assume that cannabinoid receptors are involved in key pathways in cancer. Most of our mechanistic information on the role of the ECS and endocannabinoids in cancer comes from preclinical studies.

#### THC—in vitro

THC appears to prevent proliferation in certain cancer cells, with THC’s effect on cancer cell growth and proliferation varying depending on the type of cancer cell. In breast cancer, for example, it appears to be at least partially dependent on CB receptor expression, where some studies show an inhibition of cell growth and proliferation^182^^–^^184^ with administration of THC, while others show increased proliferative effects^185^ when CB receptor expression was low. In addition to reducing proliferation, THC also appears to induce apoptosis of tumor cells, *via* increasing caspase-3.^186^

#### CBD—in vitro

CBD appears to have anti-proliferative and pro-apoptotic effects, resulting in inhibiting cell migration, invasion, and metastasis.^187^ A recent review by O’Brien^188^ covers this in-depth, but in summary, animal models demonstrate inhibition of tumor progression in a number of cancers, including brain, breast, lung, prostate, and colon cancer, and melanoma.^189^ The most likely mechanism of action is *via* modulation of reactive oxygen species (ROS), endoplasmic reticulum (ER) stress, and immune modulation. Reactive oxygen species are a type of unstable molecule that contains oxygen and that easily reacts with other molecules in a cell. Manipulation of the levels of ROS appears to be pivotal in determining if a cell proliferates or undergoes cell death.^190^ In certain cases, such as in glioblastoma, CBD appears to increase the rate of ROS formation in tumor, but not healthy cells, and, similar to THC, increases the expression of caspase-3, leading to cell death.^191^ Likewise, the ER is an important organelle that plays a critical role in post-translational modification, folding of proteins, and quality control. This quality control occurs *via* the unfolded protein response (UPR), occurring when there are too many unfolded/misfolded proteins accumulating. The UPR temporarily halts the protein synthesis and attempts to fold or repair these proteins. If this is unable to be corrected, then there is an increase in C/EBP homologous protein (CHOP), which in turn causes cell apoptosis. Increases in ER stress *via* increased ROS appears to lead to cell apoptosis. What is still unclear is whether CBD-induced ER stress and ROS generation are mediated through activation of the CB1, CB2, TRPV1, or other channels.^187^

### Cancer and cancer treatment symptom management

Most human studies have focused on either the side effects of cancer treatment, such as chemotherapy-induced nausea and vomiting (CINV), or of the cancer itself (such as weight loss and pain). Most evidence is looking at synthesized trans-Δ9-tetrahydrocannabinol, such as Dronabinol, or a CBD:THC-containing extract such as nabiximols, which is extracted from the cannabis plant itself. There is long-standing evidence dating back to the 1970s demonstrating that THC is an effective treatment for CINV,^192^ however, more recent analyses have noted that while cannabinoids are superior to placebo in reducing CINV,^193^ many of the comparisons are not against modern anti-emetic treatment regimens.^194^ Therefore, while clinicians do report significant benefits for cannabinoids in CINV,^195^ firm conclusions that it is an effective and safe anti-emetic cannot be drawn, especially for orally delivered cannabinoids.^196^ Cannabis has long been known to stimulate the appetite, often colloquially referred to as “the munchies.” There is some evidence that THC-containing smoked cannabis does increase calorie intake in healthy adults by around 40%, mostly due to increased snacking between meals, leading to increased body weight.^197^ Unfortunately, while THC-containing extracts such as dronabinol appear to increase appetite, their ability to increase body weight appears to be less effective than other treatments such as megestrol.^198^ Finally, there have been studies looking at the effect of cannabis on chemotherapy-induced peripheral neuropathy. While promising, most of the evidence is in animal models,^199^ with only one small trial in 16 humans that showed some promising reductions in neuropathic pain when taking Nabiximols^200^; however, no fully powered RCTs have been undertaken to confirm this. There currently is no evidence for a benefit for nabiximols in addition to opioids in non-neuropathic cancer pain.^201^ To date, there have not been any high-quality trials comparing whole plant extracts to either placebo or other treatments for most cancer-related outcomes.

#### Brain tumours

Preliminary evidence is emerging that demonstrates the potential benefits of medicinal cannabis for glioblastoma (GBM) treatment in humans. One double-blind RCT in people with GBM (n = 21)^202^ found those who had nabiximols + temozolomide (TMZ) had a higher one-year survival rate (83%) than those in the placebo + TMZ group (44%). While the nabiximols group had a higher rate of adverse events, having a greater rate of both severe adverse events and more serious adverse events, no interaction between the nabiximols and TMZ was observed. A larger RCT of 88 participants with high-grade glioma found a nightly dose of THC-containing medicinal cannabis products (THC:CBD ratio of either 1:1 or 4:1) improved quality of life, sleep, and functional well-being.^203^ There is some evidence CBD may also assist with managing refractory seizures due to primary brain tumors. This case report included three patients with epilepsy caused by brain tumors and found improvements in seizure severity in all three, while two of the three subjects showed an improvement in seizure frequency.^204^ Dosage of CBD seems to be important, with previous evidence showing a strong correlation between CBD dosage, plasma levels, and seizure control.^205^ While the current evidence on cannabis for GBM is promising, further research is needed to fully understand the impact of various medicinal cannabis products in this population.

### Neurological disorders

A number of neurological disorders, including amyotrophic lateral sclerosis (ALS), Parkinson’s disease, Alzheimer’s disease, Huntington’s disease, Tourette’s syndrome, multiple sclerosis(MS) and epilepsy all have potential therapeutic targets for cannabis or cannabinoids^206^^–^^208^ *via* modulation of cannabinoid receptors and other non-cannabinoid receptors such as GPCRs. As with cancer, most clinical studies have not examined whole plant consumption but instead mostly focus on cannabinoid-based medications such as dronabinol and Nabiximol. For a broader overview, the authors recommend the reviews by Lacroix and colleagues^208^ and Elliot and colleagues^209^ as a starting point.

### Parkinson’s disease

Parkinson’s disease (PD) shows evidence that the endocannabinoid system undergoes a significant rearrangement after dopamine depletion in both animal models of PD, and in humans, where specific involvement of CB1 and CB2 receptors seems to be involved in regulating motor behavior.^210^ Cannabis has been thought to be a potential therapeutic because of its neuroprotective, antioxidant, and anti-inflammatory properties, which may reduce symptoms and potentially slow progression of PD.^211^. Some cross-sectional^212^ and observational studies^213^ have suggested potential benefits of cannabis for PD for both motor and non-motor symptoms, in particular reductions in tremor, rigidity, bradykinesia, sleep, and pain. However, these significant changes are yet to be supported by high-quality RCTs. To date, multiple systematic reviews have found no strong evidence for cannabis improving overall symptoms of PD when looking at high-level evidence.^210^^,^^214^ This is likely to be at least partially due to the fact that most RCTs are for a short term, between 4 to 6 weeks, while observational studies show that most of the benefit does not appear to occur until after 3 months of usage.^213^ It’s important to note that some participants in one of the RCTs did not reach the target dosage due to THC-related side effects.^215^ Future clinical trials should include a longer treatment period to determine what benefits may occur with regular consumption and also look at the potential benefits of CBD-only products, as these may have less side effects compared to THC-containing products.

#### Huntingtons

Mouse models demonstrate that the ECS is involved in the pathogenesis of Huntington’s disease. For example, CB1 receptors progressively lose their functionality in early-stage Huntington’s disease, which may increase vulnerability to cytotoxic stimuli and cellular damage.^216^^,^^217^ THC and CBD may have a role in the management of Huntington’s disease through their neuroprotective and antioxidant properties, both of which contribute to delaying disease progression.^218^ A recent systematic review, which included three RCTs on Huntington’s disease, found varied results.^219^ One study (n = 44) demonstrated improved symptoms with nabilone compared to placebo across a range of motor and non-motor symptoms.^220^ However, two studies found no improvements with medicinal cannabis despite having substantial doses of THC in one study and CBD in the other. A double-blind randomized cross over trial (n = 26) found no difference between Sativex(®) in a dose of up to 32 mg THC/30 mg CBD per day compared to placebo on motor, cognitive, behavioral, and functional scores over a 12-week period.^221^ Similarly, a small (n = 15) double-blind crossover trial found a 6 week course of CBD (avg. dose 700 mg/day) was not significantly different from placebo with regard to chorea severity.^222^

#### Tourettes syndrome

Preclinical research suggests that the ECS is dysregulated in Tourette’s Syndrome. (TS) as demonstrated by a seven-fold increase in 2-AG^223^^l^, while CB1 receptors that are located in the CNS are thought to be impaired in those with TS.^224^ An overactive dopaminergic system is one of the most consistent neurochemical abnormalities observed in TS.^224^^,^^225^ Therefore, the ECS may play an inhibitory effect on the overactive striatal dopaminergic system observed in.^226^ Cross sectional data and case reports suggest improvements on tic severity following cannabis consumption in adolescents^227^ and adults^228^^,^^229^ with TS. A recent systematic review of nine studies found cannabis was associated with a significant reduction in tic severity and urgency.^230^ More recently, a small pilot double-blind randomized controlled crossover trial (n = 12)^231^ found no difference between a vaporized single 0.25 g dose of THC 10%, balanced THC/CBD 9%/9%, CBD 13%, and placebo on the Modified Rush Video-Based Tic Rating Scale (MRVTRS). However, the 10% THC product produced a significant effect on tic urge and distress.

#### Multiple sclerosis

Using animal models of MS, cannabinoids demonstrate activation of CB1 receptors, which in turn inhibits other neurotransmitters such as glutamine and decreases neuronal excitability by the activation of potassium channels,^207^ which can reduce spasticity, a common symptom in MS. A recent review of systematic reviews, including the results of 32 studies that included THC, CBD, THC:CBD formulations, pharmaceutical cannabinoids (dronabinol and nabilone), smoked *C. sativa* plant material, and oral cannabinoid extracts, found evidence that cannabinoids reduced pain or painful spasm.^232^ Similar evidence was also found by the authors for reducing spasticity, with better evidence for THC:CBD formulations; however, improvements in spasticity were dependent on the scale used, with patient-reported scales demonstrating greater benefit.^232^ Outcomes with less convincing evidence include changes in bladder function, ataxia, tremor, and sleep.

#### Epilepsy

CBD was thought to have therapeutic potential because GPR55 receptor expression in the hippocampus is increased in epilepsy^233^ and CBD may help control epileptic seizures by modulating neuronal excitability *via* GPR55 receptor antagonism.^234^ By the blocking of GPR55 receptors, CBD mobilizes the influx of intracellular Ca^2+^, leading to decreased release of excitatory neurotransmitters and thus reduced seizure activity.^235^

Under normal conditions, CB1 receptors play an important role in regulating neuronal activity and neurotransmission. Animal models demonstrate that CB1 receptor expression is increased in epilepsy.^236^ This may suggest either (i) endogenous adaptations aimed to control neuronal hyperexcitability in epilepsy or (ii) pathological alterations that facilitate neuronal hyperexcitability.^237^

CB1 receptor agonists may have an anticonvulsant effect in epilepsy; however, the evidence is mixed.^238^ CB1 receptor agonists, including THC, are also limited by their narrow therapeutic window and psychoactive side effects.^239^ One way to address this is through the use of low-dose CB1R agonists. One study suggested that CB1R agonists may produce an anticonvulsant effect at low doses. Conversely, they may have a proconvulsive effect through TRPV1 channels at high doses.^240^

Although CBD has a lower affinity for CB1 receptors than THC, it still may have a therapeutic effect for epilepsy through its action on these receptors. CBD may work *via* negative allosteric modulation of CB1 receptors.^108^ Rather than binding to the orthosteric site, CB1 receptor allosteric modulators work by binding to small molecules or proteins to affect receptor activity.^241^ Because of this, negative allosteric modulators may reduce the potency of the CB1 receptor agonists and thus the likelihood of their undesirable psychoactive side effects. Certainly one study found that CBD reduced the efficacy and potency of THC and 2-AG.^108^ Further research is required into this unique “antagonist of agonists” effect of CBD and negative allosteric modulators for epilepsy. Their use may prove useful in ensuring the therapeutic benefits of THC while regulating their unwanted proconvulsive and psychoactive side effects.

Both THC and CBD appear to have an anticonvulsant effect. THC appears to work *via* agonism of CB1 and CB2; however, the mechanism(s) of action for CBD are still at least partially unclear, as they do not demonstrate the same properties at CB1 and CB2.^242^ The anticonvulsant activity of CBD may involve blocking reuptake of ANA, activation of TRPV1 receptors, and modulation of various other receptors and compounds, including adenosine receptors, voltage-dependent anion selective channel protein (VDAC1), and TNFa release.^243^ Both open-label, and randomized controlled trials in children with Dravet Syndrome and Lennox–Gastaut Syndrome,^209^ and in a mixed population of children and adults^244^ have demonstrated benefit for CBD in reducing seizure frequency. Evidence for THC-containing products is currently less clear and mostly relies on case reports and self-reported changes,^245^ and unlike CBD, is associated with substantial adverse events.

### Chronic non-cancer pain

This is a broad area, covering a range of conditions including pelvic pain, headache, migraine, chronic neuropathic pain, chronic musculoskeletal pain, and menstrual pain. There have been RCTs on neuropathic pain, chronic prostatitis/pelvic pain, carpal tunnel syndrome, and back pain, and non-randomized studies on pelvic pain/menstrual pain. Overall the quality of evidence is either low or very low, and this limits the ability to determine the effectiveness of various cannabinoid medicines in this population.^246^ However, given the difficulties in managing chronic pain, current clinical practice guidance recommends offering a trial of non-inhaled forms of cannabis or cannabinoids in people with chronic pain that does not respond to standard treatment.^247^

### Side effects and clinical considerations in medicinal cannabis

A list of the common and rare adverse side effects associated with cannabis-based medicines has been outlined in Table 1 below, adapted from MacCallum and Russo.^100^

**Table 1.** Side effects associated with cannabis-based medicines

Most common	Common	Rare
Drowsiness/fatigue Dizziness Dry mouth Cough, phlegm, bronchitis (smoking only) Anxiety Nausea Cognitive effects	Euphoria Blurred vision Headache	Orthostatic hypotension Toxic psychosis/paranoia Depression Ataxia/dyscoordination Tachycardia Cannabis Hyperemesis Diarrhoea

It should be noted that the majority of the side effects noted in Table 3 are associated with THC. In relation to CBD, a Therapeutic Goods Administration (TGA) report on the safety of low-dose cannabidiol published in 2020 noted that the most common side effects reported were diarrhea, changes in weight or appetite, tiredness, sedation, sleep disturbances, infection, anemia, and elevated transaminase levels.^117^^,^^125^ The majority of evidence specific to the safety of CBD and potential side effect profile has investigated doses of 2 mg/kg/day (@120 mg per day), with minimal data investigating lower doses of 1 mg/kg/day, and with regard to elevated transaminase levels and hepatic injury, this has largely been observed at doses of 10–20 mg/kg/day (@620–1240 mg in adults); however, no evidence of abnormal liver function tests or hepatic injury was observed at the dose range of 60 mg of CBD per day.^117^

In a recent scoping review of systematic reviews investigating the benefits and harms of medical cannabis (mainly THC), adverse effects were reported in most reviews comparing cannabis with placebo, with serious adverse effects reported in 36% of reviews and 51% reporting minor adverse effects.^248^ Of the serious adverse effects, these included psychotic symptoms, severe dysphoria, seizure, and urinary tract infection, while the most commonly reported minor adverse events included drowsiness, dizziness, dry mouth, and nausea.^248^ Withdrawals due to adverse events in this scoping review were reported in 37% of reviews.^248^

Cannabis, particularly with frequent, long-term, or excessive use, can cause potentially negative long-term health outcomes, even when used medically. While many people use cannabis for medicinal or recreational purposes with few issues, there are potential risks, especially depending on the dose, method of consumption, individual health factors, and the variety and phytochemical composition of cannabis used.

### Schizophrenia

There has been consistent evidence over the last 40 years that there is a relationship between schizophrenia and cannabis use.^249^ Longitudinal data is supportive of a causal relationship,^249^^,^^250^ and a recent 2016 meta-analysis identified that there is an increased risk of psychosis in ultra-high-risk adolescents with a DSM-diagnosed cannabis use disorder.^251^ Of particular importance in this discussion is that the majority of studies have been conducted on participants consuming illicit, non-quality-assured cannabis products, which are typically bred to have higher THC concentrations, and it appears that it is the THC that is of concern within this cohort. The psychotropic effects of THC may mimic the presentation of psychotic symptoms, namely sensory alteration, paranoia, euphoria, and hallucinations,^249^^,^^252^ with laboratory-based experiments demonstrating that patients with schizophrenia appear to be more sensitive to the psychosis-inducing effects of THC *versus* healthy controls.^249^^,^^253^ Conversely, CBD has minimal deleterious psychotropic or impairing effects, with evidence showing it may actually be beneficial in treatment-resistant schizophrenia,^249^^,^^254^^,^^255^ albeit more clinical evidence is necessary.

### Cannabis hyperemesis syndrome

Cannabis hyperemesis syndrome (CHS) is a relatively new medical diagnosis, characterized by recurrent episodic nausea, emesis, abdominal pain, and subsequent dehydration in people that have used cannabis.^256^^,^^257^ Typical presentation is in young adults with a long and chronic history of cannabis use, often over 10 years.^256^ The pathophysiology of CHS is poorly understood, but an unusual and defining characteristic in the case report literature to reduce nausea and vomiting by patients is compulsive immersion in hot water, be that shower or bath. This can be up to 20 times per day and/or for prolonged periods of time. This compulsive behavior to reduce symptoms has been described in all but 2 reported cases, being considered a pathognomonic feature of CHS.^257^

### Pregnancy and lactation

The ECS has a fundamental role to play in various aspects of neurodevelopment as well as peripheral organogenesis. CB1 and CB2 receptor mRNA has been characterized by day 11 of gestation in rat models,^258^ and by week 14 in human embryos,^259^ with increasing concentrations of CB1 receptors in the frontal cortex, hippocampus, and cerebellum occurring by week 19.^260^ There is also a role for the endocannabinoids themselves, with AEA being present in very low levels during the early development period,^261^ and slowly increasing throughout gestation.^262^ Conversely, 2-AG levels appear to be much higher than AEA in early pregnancy, similar to those in adult brains, and peak very soon after birth.^262^ This uneven distribution of CB1 receptor expression in the brain during early phases of development, along with the fluctuations in expression as development progresses, combined with the changes in levels of circulating endocannabinoids, suggests that the ECS may play a vital role in the maturation of the nervous system.

Animal models support that the ECS, and especially CB1 receptors, is involved in various aspects of neural development and neuronal identity acquisition, including neuronal migration, synaptogenesis, axonal elongation, migration and connectivity, glia formation, and neural stem cell proliferation and differentiation.^263^^–^^265^. The involvement of the ECS in neural development is supported by human studies demonstrating neurological effects in offspring that have received cannabis exposure in utero, including increased aggression and attention in young girls at 18 months of age,^266^ a decrease in short-term memory at 3 years of age^267^ and lower verbal reasoning scores and deficits in short term memory at age 6.^268^ While a long history of cannabis consumption during pregnancy has been noted, there is a lack of robust safety data.^100^^,^^269^ A recent 2020 review concludes that the literature available suggests that no amount of cannabis use in pregnancy and lactation is safe and that it has the “potential for adverse maternal, foetal and long-term childhood development”.^270^

Additionally, the American College of Obstetricians and Gynecologists, the American Academy of Paediatrics, the Food and Drug Administration (FDA), and the US Centres for Disease Control and Prevention all state that people should avoid cannabis use during pregnancy and while breastfeeding.^271^ In Australia, both the Queensland government and the TGA mirror such recommendations, stating that products containing THC are generally not appropriate for patients who are pregnant, planning on becoming pregnant, or breastfeeding.^272^ The use of cannabis while breastfeeding remains contentious, with limited and inconsistent evidence about its effects on breast milk composition and the infant. A small PK study (n = 8) found that low concentrations of THC were detected in breast milk up to 4 hours after inhalation of 0.1 g cannabis (23.18% THC). These concentrations were such that an exclusively breastfed infant would ingest approximately 2.5% of the maternal THC dose.^273^ Similarly, a prospective study of 20 breastfeeding mothers found that THC and CBD accumulate in breast milk.^274^ A recent cross-sectional study also found that cannabis may alter the macronutrient profile of breast milk; breast milk samples with detectable cannabis metabolites had greater levels of protein and lower fat levels than samples without detectable cannabis metabolites.^275^ While these studies suggest potential alterations to breast milk composition with cannabis, the long-term effects of exposure to THC and CBD on the developing brain are unclear, and research is needed into the long-term effects of cannabis exposure during breastfeeding.

### Cannabis and the cardiovascular system

Clinical guidance on the use of medicinal cannabis has indicated that cannabis preparations should be used cautiously in those with unstable cardiac conditions such as angina pectoris, due largely to the ability of THC to cause tachycardia and possible hypotension.^100^ Further evidence highlights that consumption of higher doses of cannabis can cause postural hypotension that can lead to dizziness and syncope.^276^^,^^277^ The mechanism behind the increased heart rate associated with cannabis use is believed to be related to vasodilation causing reflex tachycardia.^277^^,^^278^ Additionally, cannabis use has a reported arrhythmogenic activity, with evidence suggesting a 20–100% increase in heart rate, which can last up to 2–3 hours.^277^

Moreover, a systematic review of case reports has identified that cannabis use may be associated with atrial fibrillation,^278^ with other case report evidence reporting ventricular tachycardia in a heart transplant patient and ventricular fibrillation^277^ being observed; however, large-scale evidence of this in clinical trials of quality-assured and standardized medicinal cannabis products is scarce.

Some of the proposed mechanisms for cannabis causing cardiovascular events include autonomic dysfunction, endothelial damage, increased sympathetic activity, angiopathy, and higher than normal carboxyhemoglobin levels.^277^ While growing case reports/series of acute coronary syndrome (i.e., myocardial infarction) and cannabis use have been reported worldwide, this has been predominantly in otherwise healthy, young, male cannabis consumers. Cannabis smoking has been associated with an increased risk of myocardial infarction 4.8 times over baseline within 1 hour of use^279^; however, in a long-term 18-year follow-up study, there was no statistically significant association between cannabis use and mortality.

### Cannabis and the cerebrovascular system

Akin to the cardiovascular system, research into the impact of cannabis on the cerebrovascular system largely focuses on recreational and illicit use; such research is also early and lacks the depth required to draw accurate findings but is important to mitigate risk. Evidence exists that proposes a 17% increase in risk of hospitalization due to acute ischemic stroke amongst recreational cannabis users (independently associated) between the ages of 18–54 years^280^ and a temporal link has been reported in several case studies with no other apparent causation.^277^^,^^281^

A prospective study in 48 young patients demonstrated that cannabis use was associated with multifocal angiopathy resulting in ischemic stroke,^282^ and numerous underlying mechanisms potentially contributing to stroke after cannabis consumption, including hypotension, vasculitis, vasospasm, and cerebral vasoconstriction syndrome.^277^^,^^281^ Other proposed mechanisms include cerebral auto-dysregulation, cardioembolism, increased carboxyhemoglobin levels, and cerebral artery luminal stenosis.^277^

### Cannabis and cognitive effects

The cognitive effects of cannabis, particularly associated with inhaled high-potency THC-dominant chemovars, are well documented.^283^ Changes to functional and structural integrity, memory, learning, and increased anhedonia have been documented,^284^ with inconsistent evidence specific to attention, learning, executive function, motor and perceptual motor function, sleep, and forgetfulness/retrieval of information also being noted.^285^^,^^286^ Further evidence supporting these cognitive effects was highlighted in a significant review conducted by the National Academies of Science, Engineering, and Medicine, which highlighted that moderate evidence exists of a statistical association between acute cannabis use and impairment in learning, attention, and memory domains.^91^

Specific to intelligence, measured by the Intelligent Quotient (IQ), it has long been touted in population-based drug-specific educational strategies that cannabis use reduces human intelligence by damaging or killing brain cells (i.e., neurons). While consumption of cannabis, particularly those chemovars high in THC, can cause a decreased function in short-term memory (as discussed above), these effects are usually short-lived and resolve with cessation. A 2016 review of two longitudinal twin studies conducted by Jackson *et al* published in the Proceedings of the National Academy of Sciences found that cannabis-using twins failed to show significantly greater IQ decline relative to their abstinent siblings, suggesting that observed IQ declines are more attributable to familial or other factors.^287^

### Cannabis-associated drug interactions

The evidence of cannabis causing drug interactions is still an evolving area of research, and this section aims to capture the available data for pharmacokinetic and pharmacodynamic interaction types. Currently, the majority of evidence relating to cannabis and drug interactions is based largely on *In-vitro* and *In-vivo* studies,^288^^,^^289^ with the relevance and impact of such experimental findings still needing to be elucidated to determine the extent of clinical impact.

Pharmacodynamic (PD) interactions are defined as when drugs (including herbal medicines and supplements) can impact or modify each other’s pharmacological effects directly.^290^ Essentially, pharmacodynamic interactions are concerned with the biochemical and physiological effects the drug(s) have on the body and include the relationship between drug concentration and magnitude of drug effects.^291^ THC exhibits more noted potential PD interactions than CBD, particularly around pharmaceutical agents related to analgesia and sedation, and other non-prescribed depressants such as alcohol. Evidence exists of individuals (n−21) who vapourized cannabis and experienced increased analgesic effects of opioids despite no alteration in plasma opioid levels.^292^ Interestingly, studies have also suggested that medicinal cannabis preparations reduce the consumption of opioids,^293^ with another study also demonstrating this in the endometriosis cohort.^294^ In relation to alcohol, low-dose alcohol was found to increase the blood levels of THC, which may explain the reduced performance when mixing THC-based cannabinoid products and alcohol, and is why alcohol use during the trial is highlighted in the inclusion criteria.

In an animal model of neuropathic pain, it was found that THC exhibited a synergistic interaction with gabapentin, whereby gabapentin improved the therapeutic window of THC while also enhancing its anti-allodynic activity.^295^ Similarly, additive effects of THC with CNS depressants and antihistamines are also possible, as in an increase in tachycardia with concomitantly administered tricyclic antidepressants, sympathomimetics, and stimulants.^296^ Both types of additive PD interactions are an important clinical consideration.

Pharmacokinetic (PK) interactions, on the other hand, are much less easy to predict. Due to the fact that PK interactions are largely unpredictable until observed in the clinical literature, they are of far greater clinical concern, particularly for medications that are categorized as narrow therapeutic index (NTI),^291^ as outlined in Table 2.

**Table 2.** Common narrow therapeutic index pharmaceutical drugs

Common narrow therapeutic index drugs	
Anti-arrhythmics (e.g., quinidine, disopyramide)	Monoamine oxidase inhibitors (e.g., selegiline, phenelzine)
Hypoglycaemics (e.g., insulin)	Antineoplastics (e.g., methotrexate)
Antiepileptics/anticonvulsants (e.g., phenytoin, valproic acid)	Opioid analgesics (e.g., Fentanyl, hydromorphone)
Immunosuppressants (e.g., cyclosporine)	Barbiturates
Mood-altering drugs (e.g., lithium carbonate)	Theophylline (1,3-dimethylxanthine)
Anti-HIV drugs (e.g., saquinavir)	Cardiac glycosides (e.g., digoxin)
Tricyclic antidepressants	Blood thinners (e.g., warfarin)

Adapted from Sinclair 2014.

Other factors are important considerations when it comes to PK interactions, such as age-related changes to organ function in the elderly or very young, inter-individual variability, comorbidities, gender, body composition, pregnancy, and organ function. All can impact drug responses and should be carefully considered when assessing potential drug interactions, whether they are of a pharmacokinetic or pharmacodynamic action.^291^

CBD is metabolized via CYP3A4, which is the same isoenzyme that 60% of clinically prescribed drugs are also metabolized through.^297^ CYP2C19 is also another isoform through which extensive metabolism occurs. Additionally, CBD can inhibit CYP2C19, CYP2D6, and CYP2C9 and may also inhibit certain CYP3 family members.^297^ Ketoconazole, ritonavir, itraconazole, and clarithromycin inhibit CYP3A4, which could potentially lead to increased levels of CBD in serum when concomitantly consumed.^297^ Conversely, CBD may increase serum levels of sildenafil, cyclosporine, antihistamines, statins, anti-retrovirals, and haloperidol.^297^ A list of metabolic drug interactions related to CBD has been described below in Table 3.

**Table 3.** Metabolic drug–drug interactions between CBD and enzyme substrates, inhibitors or inducers

Enzyme	Medications involved	Outcome(s) and management recommendations
CYP3A4 substrates	Immunosuppressants, chemotherapeutics, antidepressants, antipsychotics, opioids, benzodiazepines, z-hypnotics, statins, calcium channel blockers, others	Increased risk of side effects related to substrate. Avoid co-administration, reduce substrate dose, monitor for adverse effects and toxicity. Avoid prescribing cascade with new treatment for side effects.
CYP3A4 inhibitors	Strong: Protease inhibitors, ketoconazole, loperamide, nefazodone Moderate: Amiodarone, verapamil, cimetidine, aprepitant, imatinib	Increased CBD bioavailability, possible increase in risk of adverse effects. Reduce CBD dose.
CYP3A4 inducers	Strong: Enzalutamide, phenytoin Moderate: Carbamazepine, topiramate, phenobarbital, rifampicin, efavirenz, pioglitazone	Decreased CBD bioavailability, possible decrease in CBD effectiveness. Increase CBD dose.
CYP2C19 substrates	Antidepressants, antiepileptics, proton pump inhibitors, clopidogrel, propranolol, carisoprodol, cyclophosphamide, warfarin	Increased risk of side effects related to substrate. Avoid co-administration, reduce substrate dose, monitor for adverse effects and toxicity. Avoid prescribing cascade with new treatment for side effects.
CYP2C19 inhibitors	Strong: Fluvoxamine, fluoxetine Other: Proton pump inhibitors, cimetidine, ketoconazole, clopidogrel, fluconazole, efavirenz	Increased CBD bioavailability, possible increase in risk of adverse effects. Reduce CBD dose.
CYP2C19 inducers	Rifampin, carbamazepine, phenobarbital, phenytoin, St. John’s Wort	Decreased CBD bioavailability, possible decrease in CBD effectiveness. Increase CBD dose.
CYP2C8/9 substrates	Rosiglitazone, burprenorphine, montelukast, celecoxib, sulfonylureas, losartan, naproxen, phenobarbital, phenytoin, rosuvastatin, valsartan, warfarin	Increased risk of side effects related to substrate. Avoid co-administration, reduce substrate dose, monitor for adverse effects and toxicity. Avoid prescribing cascade with new treatment for side effects.

Adapted from^125^.

Furthermore, due to the high-protein-binding characteristics of CBD, it also has the potential to interact with other drugs that are similarly highly protein bound, such as warfarin, cyclosporine, and amphotericin B. Specific to CBD, the CBD-dominant product Epidiolex did cause elevation of the N-desmethyl clobazam metabolite of the anticonvulsant clobazam at doses of 25 mg/kg/day, which produced clinical effects of sedation, with noted caution suggested to be applied to other benzodiazepines and valproic acid being noted.^298^

THC and its metabolite 11-hydroxy-THC (11-OH-THC) are the main intoxicating cannabinoids associated with cannabis, whether use is illicit or medicinal. It has been stated that 11-OH-THC is equipotent, or more potent, an intoxicant as THC.^299^

THC is metabolised by P450 enzymes, predominantly CYP3A4 and CYP2C9.^296^ THC also exerts a broad inhibitory effect on CYP3A, CYP2D6, CYP2C9, CYP2C19, CYP2A6, CYP2B6, CYP1A1/2, and CYP2J2.^299^^,^^300^ In difference to CBD, THC and its metabolites have been found to be poor substrates or inhibitors of either P-glycoprotein or BCRP,^301^ but it has been found to exert a strong inhibitory effect on carboxylesterase 1 (CES1).^299^

In contrast to CBD, there is a general paucity of evidence for specific examples of PK interactions in the literature. Studies of Sativex (Nabiximols) have shown that THC bioavailability was increased by up to 27% and 11-OH-THC by 204% when co-administered with ketoconazole (400 mg over 5 days), which is a potent CYP3A4 inhibitor.^299^ Participants of this study experienced adverse events, notably impacting the central nervous system, and were posited to be caused by THC and 11-OH-THC toxicity.^302^ Conversely, when co-administered with 600 mg of rifampicin over 10 days (a potent CYP3A4 and CYP2C19 inducer), THC C_MAX_ decreased by 36% and 11-OH-THC by 87%, whilst omeprazole (40 mg over 6 days), which is a CYP2C19 inhibitor, caused no change in THC or its primary metabolites bioavailability.^302^

Given these concerns, it is interesting to note that a systematic review in 2014 determined that studies of THC, CBD, and CBN inhibition and induction of major human CYP-450 isoforms generally reflect a low risk of clinically significant drug interactions with most use, but that human clinical data is lacking.^303^ MacCallum and Russo^100^ are similarly supportive of this view, being prescribing cannabinoid physicians, positing that there is no drug that cannabis cannot be used with, and that “*pertinent drug interaction studies*” are few, not just for major cannabinoids such as THC and CBD, but even more so for the minor cannabinoids. With the plethora of medicinal and adult-use cannabis products entering markets internationally, many containing minor cannabinoids such as CBG, THCV, CBC, and others, more research is needed to more fully understand the PK characteristics of these minor cannabinoid compounds and their potential role in drug interactions.

## Conclusion

At present, cannabis is being used in the community for both recreational and medical purposes. In the case of medical usage, it may be prescribed by a medical doctor or purchased either legally or illicitly for medical purposes such as symptom relief. Despite a long history, evidence for cannabis as a medicine is still an emerging field, and while potential mechanisms of action for a variety of conditions have been elucidated, high-quality randomized controlled trials in humans are still lacking for many conditions that cannabis is being used for. Despite popular belief, cannabis, like all other medicines, has potential benefits and harms, and long-term consumption of cannabis, even for medical reasons, may not be risk-free. In addition, consumption via modes of administration such as smoking or using a bong may increase the risk of negative health outcomes. Further research on quality-controlled medicinal cannabis is required for us to determine what benefits and risks there may be to its use as a medicine for a variety of conditions.
